# Receptor-Mediated Transcytosis of Leptin through Human Intestinal Cells In Vitro

**DOI:** 10.1155/2010/928169

**Published:** 2010-04-29

**Authors:** Philippe G. Cammisotto, Moise Bendayan, Alain Sané, Michel Dominguez, Carole Garofalo, Émile Levy

**Affiliations:** ^1^Departments of Pathology and Cell Biology, University of Montreal, 2900, Bd Edouard-Montpetit, Montreal, Canada H3T 1J4; ^2^Department of Nutrition, CHU-Sainte-Justine Research Center, University of Montreal, 2900, Bd Edouard-Montpetit, Montreal, Canada H3T 1J4

## Abstract

Gastric Leptin is absorbed by duodenal enterocytes and released on the basolateral side towards the bloodstream. We investigated in vitro some of the mechanisms of this transport. Caco-2/15 cells internalize leptin from the apical medium and release it through transcytosis in the basal medium in a time- temperature-dependent and saturable fashion. Leptin receptors are revealed on the apical brush-border membrane of the Caco-2 cells. RNA-mediated silencing of the receptor led to decreases in the uptake and basolateral release. Leptin in the basal medium was found bound to the soluble form of its receptor. An inhibitor of clathrin-dependent endocytosis (chlorpromazine) decreased leptin uptake. Confocal immunocytochemistry and the use of brefeldin A and okadaic acid revealed the passage of leptin through the Golgi apparatus. We propose that leptin transcytosis by intestinal cells depends on its receptor, on clathrin-coated vesicles and transits through the Golgi apparatus.

## 1. Introduction

Endocrine leptin is mainly produced by the white adipose tissue [1] and its primary function consists in the long-term control of food intake and energy storage [2, 3]. It is also involved in various other physiological processes, including reproduction, immunity, and cell growth [4–6]. Aside from adipocytes, epithelial chief cells of the gastric mucosa were found to be a major source of leptin [7, 8] and this secretion has been established to be exocrine being discharged into the gastric lumen to the gastric juice [7, 8]. 

Gastric chief cells synthesize and store leptin in their secretory granules which also contain pepsinogen [8]. To ensure its integrity in the proteolytic environment of the digestive tract, gastric leptin is secreted complexed to a protective binding protein that results from the cleavage of membrane-bound leptin receptor [9]. The cleavage of the membrane bound receptor generates the soluble isoform of this receptor. The complex leptin-soluble receptor is formed in the secretory granules of the chief cells and upon stimulation released into the gastric juice [9]. It is then channelled to the duodenal lumen [9]. 

At the level of the duodenal enterocyte apical membrane, leptin binds to its membrane-bound receptor triggering some signalling cascades [10, 11]. In human, three membrane-bound receptors have been cloned and identified as 219.1 (long isoform), 219.2, and 219.3 (short isoforms) [12]. Their extracellular and transmembrane domains are identical, with an intracellular domain varying in size and aminoacid sequence, suggesting that each membrane-bound receptor activates different intracellular pathways and possesses specific roles [12, 13]. They control, amongst other processes, nutrient absorption and mucus secretion [10, 11, 14]. The particular leptin soluble receptor that is generated by the proteolytic cleavage of the extracellular domain of membrane-bound ones [9] protects leptin, and increases its half-life [9]. 

Interestingly, membrane-bound and soluble leptin receptors share a similar structure, have the same affinity for leptin and their competition modulates leptin action on the intestinal wall [15]. Finally, upon internalization by the duodenal epithelial cells, leptin is transcytosed through the cells and released on the basolateral side to reach the blood stream in an intact form [16]. A similar transport of macromolecules across the intestinal epithelial cells has been previously reported for several other proteins [17–19]. 

Molecular events taking place for duodenal leptin transport have not been established. We have therefore undertaken the present study to characterize some aspects of the leptin intestinal transcytosis. Using human intestinal Caco 2/15 cells, we demonstrate that this transport across the cells is dependent upon the long isoform of the leptin receptor. We also show that as for other cell types the endocytotic process seems to be clathrin-dependent. In addition, our findings emphasize the role of the Golgi apparatus in the intracellular leptin trafficking and reveal that leptin released into the basal medium is bound to its protective soluble receptor.

## 2. Material and Methods

### 2.1. Antibodies and Chemicals

Rabbit antibodies against human leptin (A20) and human leptin receptor (H300) were purchased from Santa Cruz Biotechnology (Santa Cruz, CA). Monoclonal mouse anti*β*-actin (clone AC15), fluorescein isothiocyanate (FITC), and albumin were from Sigma-Aldricht (St-Louis, MO, USA). Monoclonal mouse antiTNG38/42 (MA3-063) was obtained from ABR (Cedarlane Laboratories, Hornby, Ont.). Secondary antibodies with fluorescent tags Alexa 488 and Alexa 568 were from by Invitrogen (Burlington, ON, Canada). The secondary antibodies antirabbit/mouse for Western blot were part of the detection kit from Roche Diagnostics (Indianapolis, IN). Protein-A-HRP (Horse Radish Peroxidase) was from GE Healthcare limited (Amersham, UK). Human recombinant leptin was purchased from R&D systems (Minneapolis, MN). Recombinant insulin was from Eli Lilly (Toronto, On, Canada).

### 2.2. Synthesis of Leptin-FITC

FITC (50 *μ*g) and human recombinant leptin (1 mg) were solubilized in 300 *μ*L of NaHCO_3_ buffer (50 mM; pH 9) and left overnight at 4°C with agitation, as previously reported [16]. The following morning, dialysis was performed in PBS (pH 7.4) for 24 hours to remove free nonbound FITC. The final concentration of Leptin-FITC in PBS was brought to l mg/mL.

### 2.3. Cell Culture

The human intestinal Caco-2/15 line was a generous gift from Dr Jean-François Beaulieu (Sherbrooke University, QC, Canada). Cells were maintained at 37°C under a 95% air and 5% CO_2_ atmosphere in a culture medium of the following composition: minimum essential medium (EMEM; Wisent, St-Bruno, QC, Can), penicillin/streptomycin (100 kU/L), MEM nonessential amino acids (0.1 mmol/L)(Gibco, Invitrogen, Auckland, NZ, USA), and foetal bovine serum 5% final (FBS; Flow, McLean, VA). 

Cells were grown in 75 cm^2^ flasks (Corning, NY, USA) until 70–80% confluence. Cell layers were then washed twice with PBS and detached using trypsin-EDTA (50 g/L-0.5 mmol/L; Gibco-BRL). After cell counting, 6-well plates with 24.5 mm polycarbonate Transwell filter (pore size 0.4 *μ*m) (Coastar, Cambridge, MA) were seeded at a density of 1 × 10^6^ cells/well. Cells were kept for 21 days postconfluency to ensure full differentiation and polarization, leading to a tight monolayer and allowing complete segregation between the upper and lower compartments of the wells. Prior to each experiment, cells were cultured in an FBS-free medium for 20 hours and electric resistance of each well was measured to confirm cell confluency and junctional tightness. At the end of the transcytosis experiment, leptin-FITC adsorbed on cell plasma membranes was removed by addition v/v of acetic acid (0.5 M)/NaCl (0.5 M) on both apical and basal mediums, followed by thorough PBS washings prior to cell homogeneization [20].

### 2.4. Small Interfering RNA

Plasmid plKO.1 was obtained from Invitrogen (Carlsbad, CA). Mission TRC shRNA target set for leptin receptor short and long isoforms was purchased from Sigma-Aldricht (St-Louis, MO, USA). Structures of the shRNA hairpins are as follows: CCGGGCCTATGAGCAAAGTAAATATCTCGAGATATTTACTTTGCTCATAGGCTTTTTG, CCGGCCAGTGTGAAAGAGAAATTACTCGAGTAATTTCTGCTTTCACACTGGTTTTTG, CCGGGCAAGATAGAAACTGCTCCTTCTCGAGAAGGAGCAGTTTCTATCTTGCTTTTTG, CCGGCGTGTCTTTACCACACAAGATCTCGAGATCTTGTGTGGTAAAGACACGTTTTTG, CCGGCCTGGGCACAAGGACTTAATTCTCGAGAATTAAGTCCTTGTGCCCAGGTTTTTG. Plasmid and insert sizes were confirmed by enzymatic digestion. Each hairpin for leptin receptor cDNA has the following structure: BamH1 site, a 21-nucleotide sense sequence, a spacer (CTCGAG), a 21-nucleotide antisense sequence, stop signal for RNA polymerase III (TTTTT) and Nde1 site. Two plasmids referred to constructs C7 and C9 were used for the invalidation procedure.

### 2.5. Lentiviral Infection

Lentiviruses were produced by cotransfecting 293T cells with packaging plasmids plp1 (5 *μ*g, gag/pol), plp2 (5 *μ*g, rev), VSV-G (5 *μ*g, envelope), and the shRNA plasmid C7 or C9 (5 *μ*g, leptin receptor) or GFP (5 *μ*g, control of successful infection). Briefly, all plasmids were mixed together in a CaCl_2_ Hepes buffer and added to cell culture medium. Following a 4-hour incubation at 37°C, the medium was removed and replaced with a fresh one containing 10% FBS. After 2 days, supernatants containing the viruses were taken, filtered through a 0.45 *μ*m filter, and frozen at −80°C until use. Viral infection was carried out for 72 hours on 2 × 10^5^ Caco-2/15 cells. They were then trypsinated and grown in T75 flasks with 10 *μ*g/mL puromycin or blasticidin 2 *μ*g/mL as selection agents for shRNA and GFP plasmids, respectively.

### 2.6. RT-PCR

RNA was extracted with Trizol buffer (Invitrogen, Carlsbad, CA, USA) as previously reported [21]. After measurement of RNA concentration at 260/280 nm, reverse transcription was carried out on 5 *μ*g of RNA using a kit from Invitrogen. PCR was performed using Fermentas kits (Burlington, On, Can) and a Biometra cycler (Montreal Biotech Inc Kirkland Canada). Primers for human leptin receptor and reference gene GAPDH mRNAs had the following sequence: 219.1 forward TTGGAAGCCCCTGATGAAA, 219.1 reverse AGCAGATAAACAAGTGAACAAAG, 219.2 forward TTGGAAGCCCCTGATGAAA, 219.2 reverse AGGTGCGCACGAGGTAGGA, 219.3 forward ATTCAATTGGTGCTTCTGTT, 219.3 reverse CATTGGGTTCATCTGTAGTG, GAPDH forward AGAAGGCTGGGGCTC, GAPDH reverse GGGCCATCCACAGTC. DNA was amplified as follows: denaturation at 95°C for 30 seconds, annealing at 52°C for 30 seconds, and elongation at 72°C for 30 seconds. For each set of primers, a saturation curve was realized to determine the linear portion of the exponential phase that was found between 28–39 cycles. Final elongation lasted 10 minutes at 72°C. All samples received loading buffer and were resolved on a 1% agarose gel with ethidium bromide (1 ng/mL final).

### 2.7. Western Blot

Cells were homogenized in lysis buffer (M-Per, Thermo Scientific, Rockford, IL, USA) containing a mix of proteases inhibitors (PMSF, pepstatin, BHT, leupeptin), sonicated and stored at −80°C until use. Protein concentrations were measured using a Biorad kit (Hercules, CA, USA). For immunoblots, samples were heated at 95°C in Laemmli buffer for 5 minutes then resolved on a 7.5% or 10% SDS-PAGE gel. Proteins were transferred onto nitrocellulose membranes in the presence of methanol. Nonspecific binding sites were saturated for 1 hour in TBS containing tween (0.1%) and milk (1%). Membranes were incubated overnight at 4°C with primary antibodies (antileptin 1/2000, antileptin receptor 1/3000, antiFITC 1/2000, anti*β*-actin 1/20000). The following day, signals were revealed using secondary antibodies bound to HRP (1/10000) and a chemoluminescent reagent (Roche Diagnostics, Indianapolis, IN).

### 2.8. Confocal Microscopy

Caco-2/15 cells were grown on glass cover slides coated with 0.1% gelatine. Upon exposure to leptin for 45 minutes and washing, cells were fixed with 4% paraformaldehyde for 20 minutes, they were permeabilized with Triton ×100 (0.1% in PBS) for 3 minutes and washed three times with a solution of glycine (5 mM in PBS). Cells were then incubated for 1 hour with foetal bovine serum (FBS 10% in PBS). Incubation with the primary antibody in FBS 10% was carried out overnight at 4°C (antiFITC 1/250, antiTGN42 1/250). The following morning, the secondary antibody was added (Alexa 488 1/500 and Alexa 568 1/500) for 30 minutes, and upon thorough washing the cells were mounted in DABCO/glycerol (v  :  v 5/50%) in PBS. Negative controls were incubated with FBS 10% alone followed by the secondary antibody in the same conditions. Slides were analyzed with a confocal microscope (Quorum WaveFX Spinning Disc in CSU10 confocal scanner unit Yokogawa mounted on an Olympus BX61WI) from Quorum technologies Inc (Ghelph, On, Canada) and images processed with Volocity software (Improvision Ltd, Waltham, MA, USA). Quantification of the staining was carried out using UN-SCAN-IT gel 6.1 software (Silk Scientific Corporation).

### 2.9. Electron Microscopy

Caco-2/15 cell cultures were fixed in 1% (v/v) glutaraldehyde in 0.1 M sodium phosphate buffer, pH 7.4, for 2 hours at 4°C. They were then washed with phosphate buffer, dehydrated in a series of graded methanol solutions at decreasing temperature (until reaching −30°C), and embedded in Epon or Lowicryl (K4M CANEMCO; St-Laurent, Canada) as reported previously [22, 23]. Semithin sections were observed by light microscopy. Thin sections of the lowicryl-embedded material were mounted on Parlodion and carbon-coated nickel grids and processed for the immunogold labeling [22, 23]. Quenching of nonspecific binding sites was carried out with glycine (0.15 mM in PBS, pH 7.4) at room temperature, followed by ovalbumin 1% in PBS. Incubation with the primary antibody was carried out overnight at 4°C (antileptin receptor 1/250). Grids were washed with PBS and incubated on 1% ovalbumin prior to the 30-minute incubation with the protein A-gold complex (10 nm). They were then washed with PBS and distilled water. Counterstaining was obtained with uranyl acetate. Control experiments were carried out in parallel by omitting the primary antibody from the labeling protocol. Cells were then examined with a Philips 410LS electron microscope (FEI Systems Canada; St-Laurent, Canada).

### 2.10. Statistical Analysis

Student *t*-tests were used to compare control and assay conditions. Results were expressed as mean ± SEM.

## 3. Results

### 3.1. Caco-2/15 Cell Monolayer as a Model for the Study of Leptin Transcytosis

**Figure 1:**
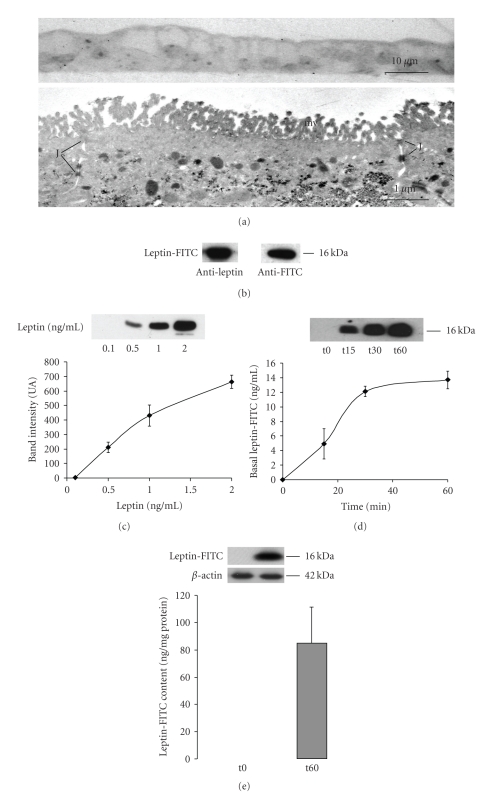
*Transcytosis of leptin through Caco-2/15 cell monolayer*. (a) Intestinal caco 2/15 cells grow as a tight polarized monolayer displaying apical microvillosities (mv) and tight junctional complexes (J). (b) Upon preparation of the leptin-FITC complex, leptin is revealed by Western blot as a single band of 16 kDa using antibodies against leptin as well as against FITC. (c) Quantification of leptin by Western blot is linear up to 1 ng/mL. (d) Leptin-FITC (10 *μ*g/mL) added to the apical compartment of Caco-2/15 cell monolayer is transported to the basal compartment along with time and appears to be saturable. (e) Leptin intracellular content was raised significantly at the end of the experiment (60 minutes). Results are expressed as mean values ± s.e.m (*n* = 4 for each experiments).

**Figure 2:**
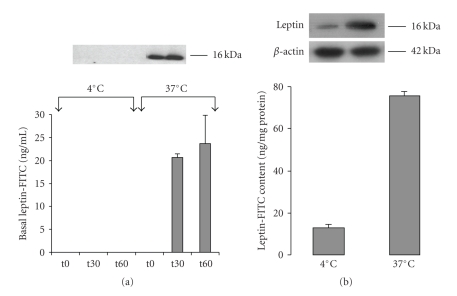
*Effect of temperature on leptin transcytosis*. Leptin-FITC (10 *μ*g/mL) was added to the apical compartment of Caco-2/15 cell monolayer which was kept either at 4°C or 37°C. Leptin concentration in the basal compartment was measured at 0, 30, and 60 minutes for both conditions. (a) Incubation at 4°C inhibits the transport of leptin to the basal compartment at all time points. (b) Leptin intracellular contents after 60 minutes of incubation showed a 85% decrease at 4°C as compared to 37°C. Bars represent mean values ± s.e.m (*n* = 4).

The Caco-2/15 intestinal cell monolayer culture demonstrates several characteristics of intestinal epithelial cells and is being widely used as a model [24]. Tightness of the monolayer was confirmed by measuring electric resistance (>600 ohms) and by light and electron microscopic examination of the monolayer itself and of the tight junctional complexes in particular (Figure 1(a)) [24]. By immunoblot, freshly formed leptin-FITC complex is recognized as a single band of 16 kDa by antibodies against leptin and against FITC (Figure 1(b)). Once well characterized, it was carefully added to the apical medium of cultures at a final concentration of 10 *μ*g/mL. The basal medium was sampled at times 0, 15, 30, and 60 minutes and cells homogeneized at 60 minutes. For each immunoblotting, a reference curve using 0.1, 0.5, 1, and 2 ng/mL of leptin was carried out to be able to quantify the leptin band density. The reference curve was found to be linear up to 1 ng/mL (Figure 1(c)). Appearance of leptin-FITC in the basal medium was evaluated as a function of time (Figure 1(d)). Leptin in the basal medium appeared in a linear fashion during the first 30 minutes with a maximal concentration at 14 ng/mL. Leptin cell content was measured at the end of the experiment (Figure 1(e)). Leptin content increased from 0 to 81 ng/mg protein in 60 minutes. The same experiment carried out at 4°C resulted in the total absence of leptin-FITC in the basal medium (Figure 2(a)) while intracellular leptin content was severely reduced (−85% compared to 37°C) (Figure 2(b)).

### 3.2. Role of Membrane-Bound Leptin Receptors in the Transcytosis of Leptin

**Figure 3:**
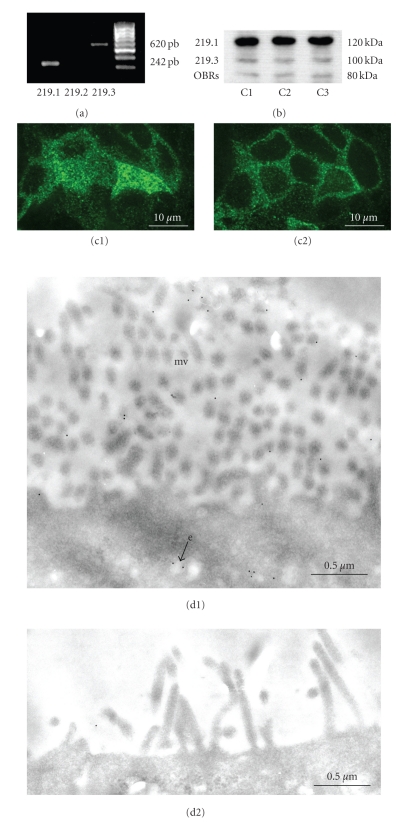
*Presence of leptin receptors in Caco-2/15 cells*. (a) Leptin receptor long (219.1) and short (219.3) isoform mRNAs are present in Caco-2/15 cells as revealed by RT-PCR analysis. (b) In three different experiments (C1, C2, C3), Western blots demonstrate the presence of the long (219.1) (120 kDa) and short (219.3) (100 kDa) isoforms of the leptin receptor, as well as that of the soluble receptor (OBRs) (80 kDa). The isoform 219.2 was not found. Confocal microscopy carried out on cell monolayer displays intense green staining for the leptin receptor on the apical pole of the cells (c1) and a more granulated and dispersed staining on more central regions of the same cells (c2). (d1) Immunogold electron microscopy confirms the presence of the leptin receptor on the apical plasma membrane particularly at the level of the microvilli (mv) and on the limiting membrane of endocytotic vesicles where gold particles are easily detected (e). (d2) Control of specificity: cell monolayers were incubated without the primary antibody showing absence of labelling by gold particles.

Caco-2/15 cells express mRNA for the long (219.1) and the short (219.3) isoforms of the leptin receptor (Figure 3(a)), while short isoform 219.2 was not detected. The presence of the long (120 KDa) and short (90 KDa) isoforms as well as the soluble isoform (80 kDa) for leptin receptor was confirmed by immunoblot (Figure 3(b)). Confocal immunohistochemical microscopy carried out on Caco 2/15 cells revealed strong labellings for leptin receptor at the apical region of the cells (Figure 3(c1)) while the signal deeper inside the same cells was of lower intensity (Figure 3(c2)). These results were confirmed by immunogold electron microscopy, which demonstrated that leptin receptors are indeed expressed on the apical plasma membrane of the Caco-2/15 cells particularly at the level of the microvilli (Figure 3(d1)) and on the baso-lateral membrane (not illustrated). They were also found on the membrane of some endocytotic vesicles (e) (Figure 3(d1)). Control for the immunocytochemical experiments resulted in an absence of labelling and very few scattered gold particles on the apical region of cells, indicating a good specificity of the labellings (Figure 3(d2)). 

**Figure 4:**
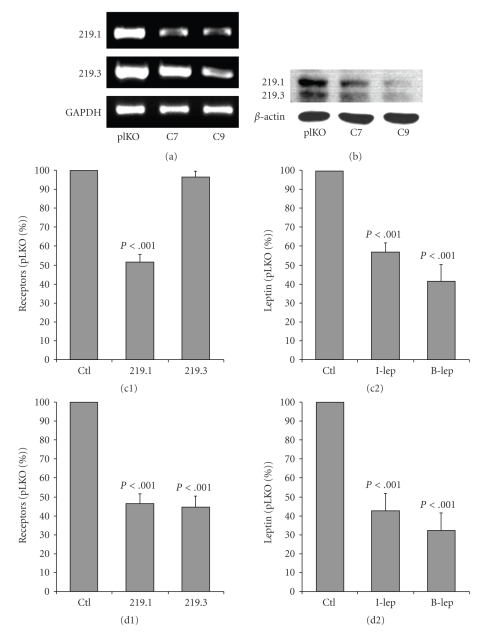
*Transcytosis of leptin in knockdown cells*. Cells were infected with lentiviruses containing plKO.1, C7, or C9 shRNA. Leptin was added to the apical medium of C7 and C9 infected cells and leptin intracellular content (I-Lep) as well as leptin concentrations in the basal medium (B-Lep) were assessed. Results of the invalidations on the long (219.1) and short (219.3) isoforms were analyzed by RT-PCR (a) and Western blot (b). (c) Infection with the C7-virus decreased expression of the long isoform (−48%) (c1) without affecting significantly the short one, and decreased I-Lep (−43%) and B-Lep (−58%) (c2). (d) C9-virus decreased both long and short isoforms, respectively by 47% and 45% (d1), and decreased I-Lep (−57%) and B-Lep (−68%) (d2). Bars represent the mean value ± s.e.m of 6 independent experiments.

To assess whether these receptors are involved in leptin transcytosis, Caco-2/15 cells were infected with lentiviruses containing a specific short hairpin RNA (shRNA). Construct C7 codes a shRNA that recognizes a sequence specific to the intracellular domain of the long receptor isoform 219.1. C9 shRNA is complementary to a sequence of mRNA common to both receptors 219.1 and 219.3. The pLKO.1 virus was generated with the empty plasmid and is used as a control. Successful invalidation of the leptin receptors was confirmed by RT-PCR (Figure 4(a)) and Western blot (Figure 4(b)). Infection with plasmid C7 led to a 48% decrease of the long isoform of the receptor (219.1) with no significant changes for the short isoform (219.3) (Figure 4(c1)). Plasmid C9 decreased the long (219.1) and the short (219.3) isoforms by 47% and 45%, respectively (Figure 4(d1)). 

Transcytosis experiments carried out on plasmid C7-infected cells revealed that leptin intracellular contents and leptin transferred to the basal medium were reduced by 43% and 58%, respectively (Figure 4(c2)). Transcytosis by plasmid C9-infected cells was decreased by 57% for intracellular leptin and by 68% for basal leptin (Figure 4(d2)). Differences in results obtained with C7 and C9 cells were not statistically different.

### 3.3. Presence of Leptin Soluble Receptor in the Basal Medium of Caco-2/15 Cell Culture

**Figure 5:**
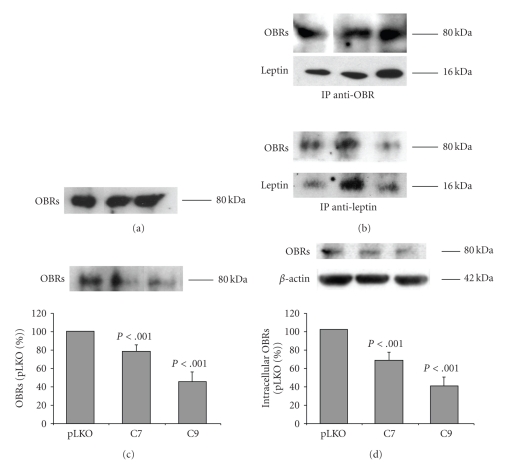
*Presence of the soluble leptin receptor (OBRs) in the basal compartment of Caco-2/15 cell culture*. (a) Detection of the soluble leptin receptor (OBRs) by immunoblot was carried out on the basal medium of 3 different experiments. (b) Immunoprecipitation of the media using the antibody against leptin receptor reveals the presence of the soluble receptor (80 kDa) associated to leptin (16 kDa); inversely, immunoprecipitation with the leptin antibody led to the same bands. Levels of soluble leptin receptor were measured in supranatant culture media (c) and in homogenates (d) from cells infected with pLKO.1, C7 or C9 virus. OBRs levels in basal media and cell homogenates were significantly decreased by C7 or C9. Bars represent the mean values ± s.e.m (*n* = 5).

We examined whether Caco-2/15 cells released the soluble isoform of the receptor complexed to leptin in the basal medium. To this end, leptin was added in the upper medium of the Caco-2/15 cell monolayer and upon 60 minutes of incubation, proteins of the basal medium were assessed by Western blot. A single band at 80 kDa was recognized by the antibody directed against the leptin receptor (Figure 5(a)) [9, 25]. Furthermore, after immunoprecipitation with the antireceptor antibody, the 80 kDa protein was found to be complexed to a protein of 16 kDa, recognized by the antileptin antibody (Figure 5(b)). Immunoprecipitation using the leptin antibody yielded the same two bands, one at 16 and one at 80 kDa (Figure 5(b)). To further evaluate the secretion of this soluble leptin receptor, intensities of the 80 kDa band in the basal media from plKO.1-, C7-, and C9-infected cells were compared. The signals were decreased 22% for C7 and 55% for C9 when compared to control pLKO.1 (Figure 5(c)). Similar measurements on infected-cell homogenates revealed a 33% and 60% decrease of intracellular soluble leptin receptor by C7 and C9, respectively (Figure 5(d)).

### 3.4. Endocytotic Pathway of Leptin

**Figure 6:**
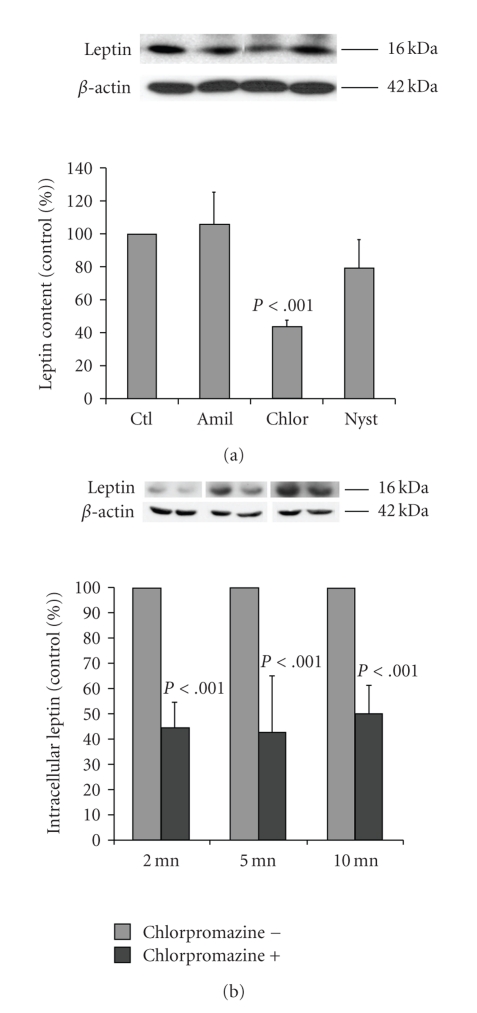
*Inhibition of leptin endocytosis*. Caco-2/15 cells were preincubated with the following inhibitors of endocytosis: Amiloride (Amil) (3 mM), chlorpromazine (Chlor) (6 *μ*g/mL), and nystatin (Nyst) (25 *μ*g/mL). Leptin was added to the apical medium for 30 minutes and intracellular leptin contents were measured. (a) A significant decrease was observed only with chlorpromazine treatment. (b) Specificity of chlorpromazine treatment was confirmed by measuring of intracellular leptin at shorter incubation times (2, 5, and 10 minutes). Mean values ± s.e.m (*n* = 4).

Caco-2/15 cells were preincubated for 30 minutes with compounds that were reported to be endocytosis inhibitors, namely, chlorpromazine which among other actions inhibits clathrin-dependent endocytosis, nystatin that inhibits clathrin-independent endocytosis, and amiloride to block macropinocytosis. Upon preincubation, leptin was added to the upper medium for 30 minutes and intracellular leptin was assessed. Amiloride (3 mM) and nystatin (25 *μ*g/mL) had no significant effect on leptin uptake while chlorpromazine (6 *μ*g/mL) decreased leptin intracellular content by 57% (Figure 6(a)). To further characterize this inhibition, cells were incubated with leptin and analyzed at shorter time (2, 5, and 10 minutes) (Figure 6(b)). Chlorpromazine was effective at all times, indicating that its effect is not mediated by an increase in leptin recycling. Also, two additional experiments were carried out. Insulin which is known to be internalized in a clathrin-dependent fashion [26] and albumin (clathrin independent absorption) [27] were tested. In the presence of chlorpromazine, insulin internalization was reduced to the same extent as leptin (42%), while albumin was only lightly affected by 11% (results not shown). 

**Figure 7:**
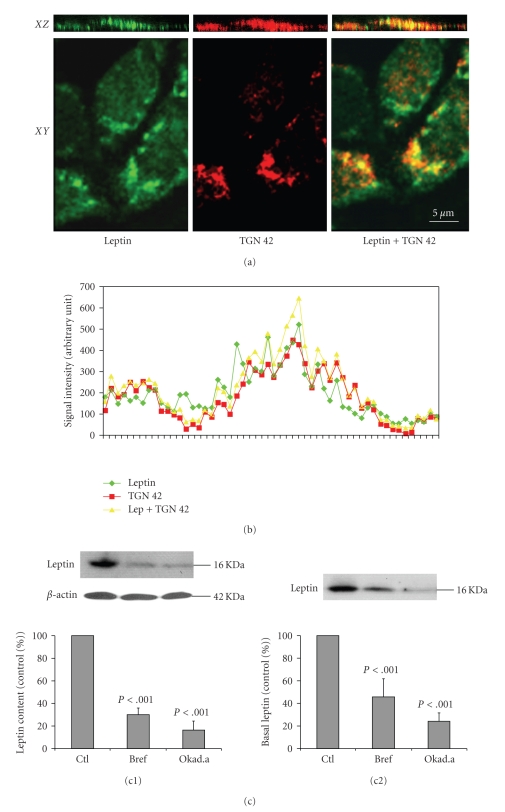
*Involvement of the Golgi apparatus in leptin transcytosis*. Caco-2 cells were exposed to leptin-FITC for 30 minutes. (a) By confocal microscopy, leptin (green fluorescence) and TGN42, a marker of the Golgi apparatus (red fluorescence), appear colocalized (yellow staining) in spots (*X* *Z* plane) or as crescent-shaped structures close to the nuclei (*X* *Y* plane). (b) Quantification of signal for leptin and TGN42 in *X* *Z* view confirmed colocalization. (c) Inhibitors of Golgi trafficking, brefeldin A (Bref) (10 *μ*g/mL), and okadaic acid (Okad.a) (0.5 *μ*M) were added to the cell medium for 30 minutes prior adding leptin. Cellular content (c1) and concentrations of leptin in the basal medium (c2) were decreased by the two agents. Results are expressed as mean values ± s.e.m (*n* = 4).

Since we previously demonstrated in in vivo studies that leptin is channelled through the Golgi apparatus in duodenal epithelial cells, we wanted to determine whether passage through the Golgi apparatus also represents a significant step along leptin transcytosis through Caco-2/15 cells. Immunocytochemistry was carried out on cells incubated for 30 minutes with leptin-FITC. Labelling for leptin-FITC revealed a positive signal within the cell cytoplasm in the form of small vesicular profiles particularly evident close to the nuclei (*X* *Y* plane, Figure 7(a)). Staining for a marker of the trans-Golgi network (TGN42) led to a red perinuclear signal that partially colocalized (yellow spots) with the leptin green signal, indicating the presence of internalized leptin in the trans-Golgi compartment (*X* *Y* plane, Figure 7(a)). Quantification of the staining intensity for leptin and TGN42 on the *X* *Z* plane confirmed their colocalization (Figure 7(b)). To further demonstrate the involvement of the Golgi apparatus in the transfert of leptin, Caco-2/15 cells were preincubated for 30 minutes with brefeldin A (10 *μ*g/mL) or okadaic acid (0.5 *μ*M) prior to leptin addition. These agents are known to break down the Golgi apparatus and alter Golgi trafficking. Brefeldin A leads to the fusion of Golgi cisternae and rough endoplasmic reticulum, efficiently blocking protein trafficking [28] while okadaic acid induces fragmentation of the Golgi apparatus [29]. Endocytosis and transcytosis of leptin under these conditions were decreased by 70% and 54%, respectively for brefeldin A, and 84% and 76% for okadaic acid, when compared to their respective controls (Figure 7(c)).

## 4. Discussion

The present study reveals the transcytotic transport of leptin across the polarized Caco-2/15 cells and highlights some of the characteristics of this transport. Leptin exposed to the apical side of the cells is internalized and transferred to the basolateral pole to be secreted into the basal medium though a transcytotic mechanism. We found that the long isoform of the leptin receptor is required in order to carry out what appears to be a clathrin-mediated endocytosis of the leptin present in the apical medium. We also show that the Golgi apparatus is involved in this intracellular transit of leptin. Leptin is eventually released into the basal medium bound to its soluble receptor. 

We have previously reported that leptin secreted by the gastric mucosa into the gastric juice in vivo and vehiculated to the duodenal lumen is captured by the intestinal mucosa, internalized, transcytosed, and secreted to be delivered into the systemic circulation [16]. Herein, we attempt to decipher certain aspects of this transport using the in vitro Caco-2/15 cell line, an efficient model of human intestinal cells [24]. Caco-2/15 cells were grown to confluency constituting a tight polarized and well-differentiated monolayer of epithelial cells as demonstrated by light and electron microscopy [24] and by the apical expression of villin and sucrase isomaltase [30]. The monolayer delimits tight upper and lower compartments, a very suitable in vitro model to study transepithelial transport [2, 31]. The tightness of the monolayer is supported by the high electric resistance of the culture monolayer and the presence of well-developed junctional complexes. 

We have demonstrated that leptin introduced in the medium of the upper compartment of the cell culture crosses the cell monolayer to reach the lower compartment, in a time- and temperature-dependent and saturable manner, suggesting the involvement of a specific transporter, as reported for other proteins [19, 31]. At 37°C, intracellular content of leptin rapidly increases; however, it was potently inhibited at 4°C (−85%), which reflects an energy-dependent mechanism. Transport through a paracellular pathway was excluded, as shown by the complete absence of leptin in the lower compartment in cold conditions (4°C). 

Receptor-mediated transcytosis is a well-established system for protein transport [32]. Studies on endothelial cells have demonstrated that leptin receptors participate in the transcytotic transport of leptin through the blood brain barrier [13, 33] and that both the short and the long isoforms of the receptor carry leptin with the same efficiency [12]. In the present study, we observed that Caco-2/15 cells also express the long (219.1) and short (219.3) isoforms of the leptin receptor, which confirms previous reports on intestinal human tissues and Caco-2/15 cell line [34–36] and these receptors are present on the apical portion of the cells at the level of the microvilli. 

Knocking-down the long isoform was effective in decreasing both endocytosis and transcytosis of apical leptin. On the other hand, the invalidation of both the short and long isoforms had no cumulative effect, suggesting that the long isoform is mostly responsible for the transport of leptin through Caco-2/15 cell monolayer. These results differ from the situation reported in endothelial cells, where the short isoform of the leptin receptor seems to be the main carrier [13]. However, in endothelial cells, the short isoform of the receptor represents 80% of all leptin receptors while for Caco-2/15 cells the long isoform of the receptor is the one to be highly expressed [13]. It must also be noted that, in renal epithelial cells, the short isoform of the receptor is the one to target the lysosomal compartment more efficiently than the long one, which indicates that the long isoform could be more involved in nondegradative intracellular and transcellular pathways [12]. 

In blood and in gastric juice, leptin circulates bound to a protective protein which is the soluble isoform of its receptor [9]. This soluble isoform of the receptor arises from the proteolytic cleavage of the membrane-bound one [37]. We found that leptin released in the basal compartment by Caco-2/15 cells is indeed complexed to this soluble receptor. The leptin complex found in the basal medium is formed by leptin and a protein recognized by the antibody against the extracellular portion of the leptin receptor which displays the expected molecular mass of the human soluble leptin receptor (80 kDa) [9, 25, 38]. Finally, the presence of the complex leptin-soluble receptor intracellularly and in the basal medium was decreased following the knockdown of leptin receptors. Our observations corroborate the origin of the soluble receptor as derived from long and short receptor isoforms by proteolytic cleavage [37]. Thus, the in vitro situation appears to mimic the in vivo one in which leptin is not secreted and does not circulate free but rather in the form of a complex bound to its soluble receptor in the blood as well as in the gastric juice [9]. 

The mechanism of leptin uptake was unravelled by the use of inhibitors of endocytosis. Chlorpromazin which among other effects is a clathrin-mediated endocytosis inhibitor inducing redistribution of AP-2 to endosomes [39]; nystatin is a cholesterol-sequestrating drug that inhibits clathrin independent endocytosis without affecting clathrin-dependent one [40], while amiloride prevents macropinocytosis [41]. In our conditions, only chlorpromazine was effective in blocking leptin uptake. Clathrin is expressed on the apical membrane of Caco-2/15 cells and is involved in the transport of transferrin [42, 43]. Furthermore, endocytosis of leptin receptors, both the long and short isoforms, is dependent on clathrin vesicles in several cell types [12, 44]. Caco-2/15 cells seem therefore to follow the classic clathrin-dependent pathway for leptin receptor endocytosis [45]. 

During transcytosis through Caco-2/15 cells, internalized leptin was found in cisternae of the Golgi apparatus, similarly to observations made in situ on duodenal tissue [16]. Involvement of the Golgi apparatus in the transcytotic pathway may be required for the formation of the complex leptin-soluble receptor found in the basal medium. Leptin, endocytosed as a free protein at the apical membrane of Caco-2/15 cells, travels to the Golgi apparatus where it must bind to newly synthesized receptor molecules prior to its release in the basal medium. This ensures protection of leptin in the basal medium as it occurs in vivo. Disruption of the Golgi apparatus by brefeldin A or okadaic acid decreased the appearance of the leptin-receptor complex in the basal medium. Several hypotheses may explain our observations. It has been reported that in Hela cells, newly synthesized short and long isoforms of leptin receptor are mostly stored in the trans-Golgi network, with low expression at the plasma membrane [46]. On the other hand, constitutive endocytosis in ligand-independent manner occurs for leptin receptor with transport to lysosomes, with a limited recycling to the cell surface [46]. In Hela-cells, 40% of both leptin receptor isoforms are internalized within 15 minutes. We can presume that in the presence of Golgi trafficking inhibitors, constitutive endocytosis in Caco-2/15 cells may suffer a severe depletion in leptin receptors at the plasma membrane, thereby limiting leptin uptake. On the other hand, okadaic acid that increased caveolae-dependent endocytosis, also decreases the clathrin one, which may explain the decreased endocytosis observed in our conditions [47]. 

In conclusion, we have shown that leptin follows a transcytotic pathway which includes clathrin-dependent endocytosis mediated by its membrane-bound receptors. After passage through the Golgi apparatus, leptin is released in the basal medium in a complex form bound to the soluble form of its receptor.
